# Effects of Site-Directed Mutagenesis of Cysteine on the Structure of Sip Proteins

**DOI:** 10.3389/fmicb.2022.805325

**Published:** 2022-04-29

**Authors:** Lin Wang, Ming-Yue Ding, Jing Wang, Ji-Guo Gao, Rong-Mei Liu, Hai-Tao Li

**Affiliations:** ^1^College of Life Science, Northeast Agricultural University, Harbin, China; ^2^State Key Laboratory for Biology of Plant Diseases and Insect Pests, Institute of Plant Protection, Chinese Academy of Agricultural Sciences, Beijing, China

**Keywords:** *Bacillus thuringiensis*, optimization of purification conditions of mutant protein, disulfide bonds, Sip1Aa, site-directed mutation, *Colaphellus bowringi* Baly, structural analysis of mutant proteins

## Abstract

*Bacillus thuringiensis*, a gram-positive bacteria, has three insecticidal proteins: Vip (vegetative insecticidal protein), Cry (crystal), and Sip (secreted insecticidal protein). Of the three, Sip proteins have insecticidal activity against larvae of Coleoptera. However, the Sip1Aa protein has little solubility in the supernatant because of inclusion bodies. This makes it more difficult to study, and thus research on Sip proteins is limited, which hinders the study of their mechanistic functions and insecticidal mechanisms. This highlights the importance of further investigation of the Sip1Aa protein. Disulfide bonds play an important role in the stability and function of proteins. Here, we successfully constructed mutant proteins with high insecticidal activity. The tertiary structure of the Sip1Aa protein was analyzed with homologous modeling and bioinformatics to predict the conserved domain of the protein. Cysteine was used to replace amino acids via site-directed mutagenesis. We successfully constructed Sip149-251, Sip153-248, Sip158-243, and Sip178-314 mutant proteins with higher solubility than Sip1Aa. Sip153-248 and Sip158-243 were the most stable compared to Sip1Aa, followed by Sip149-251 and Sip178-314. The insecticidal activity of Sip153-248 (Sip158-243) was 2.76 (2.26) times higher than that of Sip1Aa. The insecticidal activity of Sip149-251 and Sip178-314 did not differ significantly from that of Sip1Aa. Basic structural properties, physicochemical properties, and the spatial structure of the mutation site of Sip1Aa and the mutant proteins were analyzed. These results provide a molecular basis for using Sip1Aa to control Coleopteran insects and contribute to the study of the Sip1Aa insecticidal mechanism.

## Introduction

*Bacillus thuringiensis* (Bt) is a common gram-positive soil bacterium (Ichimatsu et al., 2000; Siegel et al., 2000; Tabashnik et al., 2003; Raymond et al., 2010; Lee et al., 2016). Because of its high efficiency and specificity to target pests and non-target biological safety, it is widely used as a biological insecticide in agriculture and forestry (Ichimatsu et al., 2000; Siegel et al., 2000; Tabashnik et al., 2003; Raymond et al., 2010; Lee et al., 2016). Based on differences in protein location and secretion mode, these proteins are characterized as δ-endotoxin and exotoxin (Singh et al., 2021). δ-endotoxin is a protein with a crystal structure encoded by crystal (Cry) and cytolitic genes (Swiecicka et al., 2008). Exotoxin, a metabolite secreted outside the cell during the metabolism of Bt, includes vegetative insecticidal protein (Vip) and secreted insecticidal protein (Sip) (Whalon and Wingerd, 2003; Argolo-Filho et al., 2014; Daquila et al., 2021). Limited research has been conducted on Sip. Promdonkoy et al. (2003) and Donovan et al. (2006) were the first to discover the novel Bt secretory protein Sip1A in the genomic library of strain EG2158D; its similarity to mtx3 was 46%. Liu et al. (2012) and Murawska et al. (2013) amplified Sip1A from the wild Bt strain QZL26. Our own laboratory successfully cloned a Sip gene 1,188 bp in size, from the Bt strain DQ89, with a similarity to the known Sip1A protein of 87% (Liu et al., 2012; Zhang et al., 2015). Sip protein has insecticidal activity against larvae of Coleoptera, with an LC_50_ of 1.542 μg/ml (Zhang et al., 2015). Sha et al. (2018) identified a novel Sip gene 1,095 bp in size and encoded 364 amino acids from the Bt strain QZL38. After removing the signal peptide of the first 30 amino acids, they amplified a 1,005 bp fragment encoding 334 amino acids, called Sip1Aa, which had an LC_50_ of 1.078 μg/ml for gibbon leaf beetle toxicity. Sequence analysis showed that Sip1Aa did not contain cysteine (Sha et al., 2018).

Bt is a highly effective biological insecticide, and the purification of insecticidal proteins is very important for the study of their structure and function. Geng et al. (2019) purified Vip using ion-exchange chromatography and obtained clear bands. Sattar and Maiti (2011) and Chen et al. (2014) reported a single purified protein during the screening of Vip that is effective against soap absorption insect pests. Kaur et al. (2007) optimized the purification protocol when studying Cry class proteins, obtaining a > 90% purification rate for the catalytically active SIAPN, 11% recovery rate, and ninefold purification. Okumura et al. (2006) purified pepsin-treated Cry45Aa using cation-exchange chromatography, with a concentration of 539 μg/ml, which was 27-fold higher than that of activated Cry45Aa purified using previous methods. The cytotoxic activity of the purified protein was stable in a broad pH region (pH 2.0–11.0) for 3 days, and 97% cytotoxic activity remained after incubation at 30°C for 360 min. After isolating the midgut, extracting it, and precipitating soluble proteins with acetone, Lomate and Hivrale (2010) purified proteins using gel filtration and ion-exchange chromatography. Aminopeptidase activity increased 8.95-fold after gel filtration chromatography. The purified enzyme appeared as a single band with a molecular mass of approximately 112 kDa. Zymogram analysis revealed two enzymatically active proteinases using LpNA as a substrate.

Although methods for purifying the Bt insecticidal proteins Vip and Cry class are relatively well established, the purification of Sip1Aa protein has been unsatisfactory. The insecticidal mechanism of Sip1Aa remains unclear. Protein expression is poor because of inclusion bodies and low solubility (Sha et al., 2018); hence, purification effects are unsatisfactory. Furthermore, miscellaneous bands hinder the determination of the underlying mechanism (Geng et al., 2019). Therefore, in this study, we used different strategies to improve protein concentration and purification effects.

Dimerization is a very common method of protein modification in biology. It is an important regulator of protein activity. Proteins usually form complexes through non-covalent bonds such as hydrogen bonds, ionic bonds, van der Waals interactions, and hydrophobic bonds. It is very important to explore the structure and function of proteins by constructing disulfide bonds (Arolas et al., 2009; Li et al., 2019). Li et al.’s (2021) study of disulfide bonds in non-covalent bond clusters provides accurate structural parameters and important data for the theoretical evaluation of more complex systems. Bandyopadhyay et al. (2020) studied non-covalent interactions between epinephrine and nitroaromatic compounds, and most recently, Huo et al. (2021) studied drugs that inactivate macromolecules or nanocomposites by breaking covalent and weak non-covalent bonds. The stability of a protein is key to its biological activity, and covalent cross-linked disulfide bonds are important for maintaining spatial structure. Newly introduced disulfide bonds can affect protein folding. In one study, the introduction of a disulfide bond between the C-terminus of gp120 and the immune-dominant fragment of gp41 increased the stability of the intermolecular disulfide bond (Binley et al., 2000). Xu et al. (2019) found that protein binding affinity decreased when disulfide bonds were eliminated from the PIIIA structure, whereas, Lee et al. (2016) showed that despite the SOS gp140 protein being partially denatured after being boiled in sodium dodecyl sulfate, the gp120 subunit remained linked to the extracellular domain of gp41, which suggests that the intermolecular disulfide bond was stable. Finally, other researchers have investigated the effect of the concentration of disulfide bonds on various indices (Binley et al., 2000; Cuicui and Qiyu, 2020).

Disulfide bonds are formed by the interaction of two cysteine residues. The position of cysteine in the protein space, the C-S bond, and the angle of rotation of the S-S bond affect the formation of disulfide bonds. Therefore, the selection of a suitable mutation site is key to the formation of these bonds. As software specially designed to introduce disulfide bonds, Disulfide by Design™ fully considers the factors that affect the formation of disulfide bonds in proteins. After a series of calculation rules, this software can predict with 99.4% accuracy amino acid sites capable of forming disulfide bonds (Dombkowski, 2003). Using the software, Han et al. (2009) introduced disulfide bonds into the structure of lipase to improve conformational stability and enhance thermal stability. Zhu et al. (2020) used site-directed mutagenesis to improve the activity and stability of metalloprotease. Therefore, in this study, we used the conserved structure region of Sip1Aa, Swiss-PdbViewer (Biasini et al., 2014), and Laplace conformation to design a disulfide bond mutation to study the effects on Sip1Aa-related functions.

## Materials and Methods

### Materials

#### Strains and Plasmids

*Escherichia coli* JM109 and BL21(DE3) were used as the host strains for cloning and expression studies. Vectors derived from pET-21b(+) were used to produce recombinant proteins. Plasmid pET21b containing the wild *Sip1Aa* gene was maintained in the laboratory.

#### Medium and Antibiotic

The medium used was Liquid lysogeny broth consisting of tryptone 1%, NaCl 1%, and yeast extract 1%. Briefly, 100 mg ampicillin was dissolved in 1 ml sterile water, filtered through a 0.22-μm filter membrane for sterilization, and diluted to 1:1,000.

#### Enzymes and Biochemical Reagents

Protein marker (Biomedical Technology Co., Ltd., Beijing, China) was used for sodium dodecyl sulfate–polyacrylamide gel electrophoresis (SDS-PAGE). KOD-Plus-DNA polymerase (Toyobo, Osaka, Japan) was used for all PCR reactions. An Axygen^®^ DNA Gel Extraction Kit (Axygen Biosciences, Union City, CA, United States) was used for amplification following the manufacturer’s instructions. A site-directed mutation kit (Vazyme, Nanjing, China) was used for site-directed mutation. All other reagents were homemade pure reagents.

#### Solutions and Buffers

PBS buffer (pH 7.4) consisting of KH_2_PO_4_ 2 mmol/L, Na_2_HPO_4_ 8 mmol/L, NaCl 136 mmol/L, and KCl 2.6 mmol/L was dissolved in 1 L deionized water. The binding buffer (pH 7.4) consisted of sodium phosphate 20 mmol/L, NaCl 0.5 mol/L, and imidazole 10 mmol/L. The elution buffer (pH 7.4) consisted of sodium phosphate 20 mmol/L and NaCl 0.5 mol/L. The concentration of imidazole was set at 40, 250, and 500 mmol/L. Cysteine standard solution (1 mmol/L) was used. We accurately weighed 0.017563 g L-cysteine, dissolved it in 1 mL methanol, and added ddH_2_O until the total solution reached 100 mL DTNB standard solution (10 mmol/L) was also used. We accurately weighed 0.0198175 g DTNB, prepared 50 mL solution with 50 mmol/L Na_2_HPO_4_ (pH 7.0), and stored it in a brown bottle. We prepared DTNB analytical solution (0.1 mmol/L) by combining 1 ml DTNB standard solution and 99 ml 0.25 mmol/L Tris HCl (pH 8.3) buffer solution.

#### Insects

The common leaf beetle was donated by the plant protection research unit of the Chinese Academy of Agricultural Sciences. The toxicity of the mutant protein to the third instar larvae of simian leaf beetles was determined with the leaf immersion method.

### Methods

#### Prediction of Disulfide Bonds

The amino acid sequence of Sip1Aa was submitted to the automated protein structure homology-modeling server Phyre2^1^ (Kelley et al., 2015). The resulting three-dimensional model was submitted to the ModRefiner server^2^ for optimization (Sellami et al., 2018). Swiss-PdbViewer (DeepView) from the ExPASy server was used to visualize observation.^3^ Disulfide by Design^4^ was used to analyze possible sites for disulfide bonds in the *Sip1Aa* gene sequence. A pair of amino acid sites was identified in the conservative domain. The distance between the two amino acid sites was greater than 50 bp, and they were mutated to cysteine to form a disulfide bond. The Swiss-Pdb Viewer file can be found in Additional Requirements. Predicting the feasibility of disulfide bond formation sites is shown in Supplementary Figure 1.

#### Construction of the Mutants

Methylated pet21b-Sip1Aa (Wang et al., 2020) was transferred to the competent state of *E. coli* JM109 as a template. The PCR reaction system was 50 μl, including 1 μl template, 25 μl 2 × Max buffer, 1 μl dNTP mix (10 mmol/L), and 1 μl Phanta Max Super-Fidelity DNA polymerase. To construct the mutant Sip149-251, for example, fragment A was amplified by T 149C-F and R 251C-R, and fragment B was amplified by R 251C-F and T 149C-R. The PCR procedure began with pre-denaturation at 95°C for 30 s, denaturation at 95°C for 15 s, and annealing at 72°C for 30 s at a rate of 60 s/kb lasting for 30 cycles. The elongation phase was terminated at 72°C for 5 min, and 1 μl DpnI was added to react at 37°C for 2 h for digestion. Then, a homologous recombination kit was used to recombine fragments A and B to create two cysteine mutations. The recombinant product was transferred into competent *E. coli* BL 21 and cultured at 37°C for 12 h. Positive clones were identified with the primers Sip1a-F/Sip1a-R and sequenced by Jilin Kumei Biotechnology. DNA sequences were analyzed by DNAMAN.

#### Optimization of Fermentation Conditions

The recombinant strain cloned and expressed in *E. coli* was diluted 30 times at 37°C and 220 rpm with blank medium as a control. The absorbance OD_600_ value of the bacterial solution was determined at each 6-h culture time, and each treatment was repeated three times. Based on these results, the growth curve of the bacteria was drawn.

#### Expression and Extraction of Mutant Protein

A single colony was selected and inoculated into LB tubes containing ampicillin resistance at 37°C, 220 rpm, for 12 h. This was cultured at 220 rpm for 12 h, then inoculated into 100 ml LB liquid medium with 1% inoculum at 37°C. The OD_600_ at 220 rpm was approximately 0.6. We added IPTG in a stepwise fashion (0, 1, 10, 30, 50, and 100 μl) at 16°C and 160 rpm for 12 h. The culture was subjected to 8,000 rpm for 15 min at 4°C. The bacteria were collected and cleaned with PBS buffer; this was repeated three times. Ultrasonic crushing was performed at 12,000 rpm at specified time intervals (86%, 3 s, 3 s, and 10 min). Then the supernatant was centrifuged at 4°C. Protein expression was detected via SDS-PAGE.

#### Protein Purification

Because the carrier had a tag, it could be purified using a nickel column. The nickel column was fixed with the addition of five times the column volume of sterile water, followed by eight times the volume of binding solution. The crude protein was added, followed by eight times the volume of the binding liquid, and finally eluted in a stepwise fashion.

The optimization process was as follows. Imidazole was added to the binding solution at concentrations of 10, 20, and 40 mmol/L and to the eluent at concentrations of 40, 250, and 500 mmol/L, respectively. The effects of various combinations of imidazole were revealed by SDS-PAGE. The optimal concentrations of imidazole were determined, and SDS-PAGE was used to detect the optimal purification conditions for the purified protein.

#### Determination of Free Cysteine by DTNB

Standard cysteine solution was diluted with Tris HCl buffer at 25°C. The concentrations were 0, 0.025, 0.05, 0.1, 0.15, and 0.2 mmol/L. We added 1 ml of each concentration solution to 5 ml of DTNB analysis solution, shook it well, and allowed it to rest for 10 min. Absorbance was measured at 412 nm to generate the standard curve. Next we added 1 ml of sample to 5 mL of DTNB analysis solution, shook it well, allowed it to rest for 10 min, measured the absorbance at 412 nm, and determined the concentration of free cysteine according to the standard curve.

#### Determination of the Protein Solubility of Sip1Aa and the Mutants

We determined the expression conditions of the Sip1Aa and mutant proteins by optimizing the fermentation conditions and the induced expression of the mutant protein. A single colony was selected and inoculated into LB tubes containing ampicillin resistance at 37°C, 220 rpm, for 12 h. This was cultured at 220 rpm for 12 h, then inoculated into 100 ml LB liquid medium with 1% inoculum at 37°C. The OD_600_ at 220 rpm was approximately 0.6. We added IPTG in a stepwise fashion, 50 μl in each tube at 16°C and 160 rpm for 12 h. The culture was subjected to 8,000 rpm for 15 min at 4°C. The bacteria were collected and cleaned with PBS buffer; this was repeated three times. Ultrasonic crushing was performed at 12,000 rpm at specified time intervals (86%, 3 s, 3 s, and 10 min). Then the supernatant was centrifuged at 12,000 rpm at 4°C. Protein expression was detected via SDS-PAGE.

#### Determination of Insecticidal Activity

(1) The quantitative insecticidal activity of the four purified mutant proteins was determined at six different concentrations: 50, 20, 10, 5, 1, and 0.1 μg/ml, and an appropriate amount of the liquid sample of the protein to be tested was added to the sterilized culture.
(2) Select fresh cabbage leaves (preferably of the same size) and evenly immerse them in the above protein sample solution to be tested.
(3) After the leaves are removed, they are dried on clean filter paper at room temperature (inverted once in the middle) and dispensed into culture dishes according to different concentrations, and filter paper wet with sterile water is padded into the culture dishes (which needs to be packaged and sterilized with tin box paper).
(4) Gently inoculate newly hatched ape leaf beetle larvae with a brush, inoculate 16 worms per dish, and repeat the treatment three times in each group, requiring a total of 48 worms in each group.
(5) They were maintained in a 25^°^C light incubator with a photoperiod of 14 h light, 10 h dark, and 55% ± 5% relative humidity. Periodically observe the leaf condition, check whether there is decay or drying phenomenon, whether there is water vapor condensation. After 48 h, the number of dead and live insects was counted and the corrected mortality and LC_50_ were calculated.

$$\mathrm{Adjusted}\,\mathrm{mortality}\,\mathrm{rate}\,\left(100\%\right)=\frac{\begin{array}{c} \mathrm{Survival}\,\mathrm{rate}\,\mathrm{of}\,\mathrm{controlgroup}\\ -\mathrm{Survival}\,\mathrm{rate}\,\mathrm{of}\,\mathrm{treatmentgroup} \end{array}}{\mathrm{urvival}\,\mathrm{rate}\,\mathrm{of}\,\mathrm{controlgroup}}\times 100\%$$

## Results

### Prediction of the Key Disulfide Point

**FIGURE 1:**
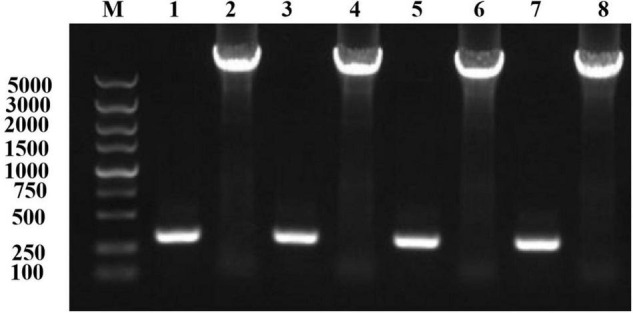
Mutation plasmid PCR amplification electrophoresis detection. Lane M, marker; lane 1, T149C-F/R251C-R; lane 2, T149C-R/R251C-F; lane 3, G153C-F/H248C-R; lane 4, G153C-R/H248C-F; lane 5, T158C-F/K243C-R; lane 6, T158C-R/K243C-F; lane 7, K178C-F/G314C-R; lane 8, K178C-R/G314C-F.

Using an online analysis server,^5^ we predicted 20 pairs of sites to form disulfide bonds. Seven pairs of sites were screened out based on the Sip1Aa conserved region, Swiss-PdbViewer (Biasini et al., 2014), and the Laplace conformation map. One pair of amino acid sites with spacing of less than 50 bp was excluded, and eight cysteine mutation sites were determined. The pET-Sip1Aa plasmid was used as the template for site-directed mutagenesis. Eight pairs of mutant primers (T149C-F/R251C-R, T149C-R/R251C-F, G153C-F/H248C-R, G153C-R/H248C-F, T158C-F/K243C-R, T158C-R/K243C-F, K178C-F/G314C-R, and K178C-R/G314C-F) were used for PCR amplification according to the instructions in the site-directed mutagenesis kit (primer sequences and amino acid sequences are listed in the additional materials). A total of 5 μL PCR product was used for agarose gel electrophoresis. The results showed that the size of the PCR amplification product was consistent with the theoretical size (Figure 1). After homologous recombination, colony PCR was used to select a single colony, and the mutant was sent to Jilin Kumei Biotechnology for sequencing. The results showed that all eight sites successfully mutated into cysteine. Primers and amino acid sequences are listed in Supplementary Figure 2.

### Detection of Newly Introduced Disulfide Bonds

DTNB was used to determine the content of free sulfhydryl in samples by colorimetry. If there are thiol compounds in the system, DTNB will turn to a yellow 5-mercapto-2-nitrobenzoic acid. As 5-mercapto-2-nitrobenzoic acid has a maximum absorption at 412 nm, the free sulfhydryl group in the sample can be determined by measuring the absorbance at 412 nm. The absorption spectra of DTNB did not interfere with the determination of the sulfhydryl group. After mixing with DTNB, absorbance was measured at 412 nm. Sip1Aa was used as a positive control, and cysteine standards of different concentrations were used as controls. The absorbance of the sample was the same as that of the standard sample without cysteine in the control group, which indicated no free cysteine in the sample, which proves that the cysteines formed disulfide bonds in a pairwise manner. The experimental results of DTNB are shown in Table 1.

**TABLE 1:** The quantity of the remaining cysteines in the mutant Sip.

	Sip1Aa	Sip149-251	Sip153-248	Sip158-243	Sip178-314	Arginine kinase
The quantity of remaining free cysteines	0	0	0	0	0	6

### Optimization of Fermentation Conditions

**FIGURE 2:**
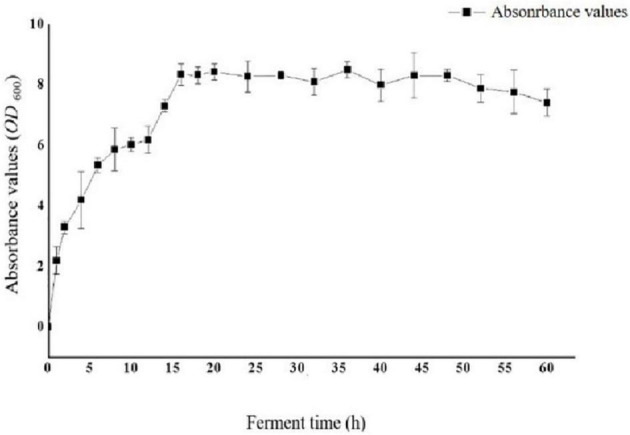
Growth curve of strain Sip1Aa.

To optimize the conditions for the expression of Sip1Aa protein, we inoculated the Sip1Aa strain into 2 × LB medium, measured the OD_600_ value of the bacterial fluid, and plotted the growth curve of the strain as shown in Figure 2. The OD_600_ value of the strain increased exponentially in the first 16 h, was stable from 16 to 48 h, and began to decrease after 48 h.

### Expression of the Cysteine Mutants

**FIGURE 3:**
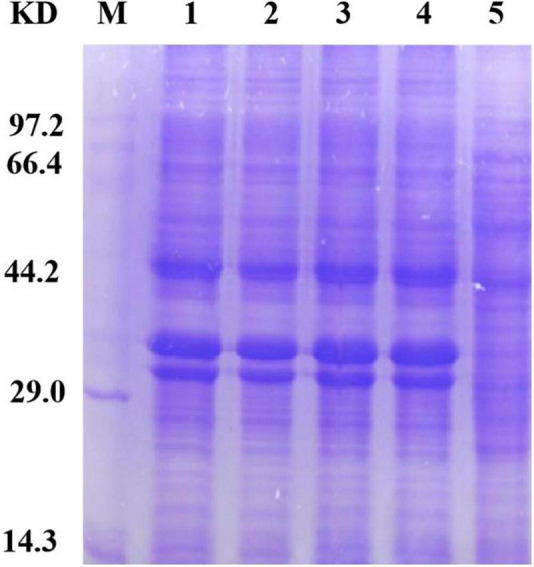
Expression of mutant proteins. Lane M, protein marker; lane 1, Sip149-251; lane 2, Sip153-248; lane 3, Sip158-243; lane 4, Sip178-314; lane 5, Sip1Aa.

To accurately induce expression of mutant proteins, we used different amounts of inducer to obtain mutant proteins with high solubility. We added 0, 1, 10, 30, 50, and 100 μl of 1 mol/L IPTG to 100 ml bacterial solution for stepwise induction. The protein expression of all four mutants was very low after the addition of 100 μl inducer. We speculate that as the amount of inducer increases, protein expression occurs more rapidly; the protein is not folded in time, and the formation of incompletely folded inclusion bodies in the precipitate leads to a low protein content in the supernatant. Protein expression was very low when a 0-, 1-, 10-, and 30-μl inducer was added; Furthermore, *E. coli* could not fully express the protein because of the low concentration of the inducer. When a 50-μl inducer was added, the four mutants were normally expressed and the solubility of the protein was enhanced, which demonstrates that the HAP mutation does not affect the expression of bacteria. The bands of the four mutants (arrow in Figure 3) were slightly thicker than that of Sip1Aa, which suggests improved protein solubility.

### Gradient Purification of Protein

**FIGURE 4:**
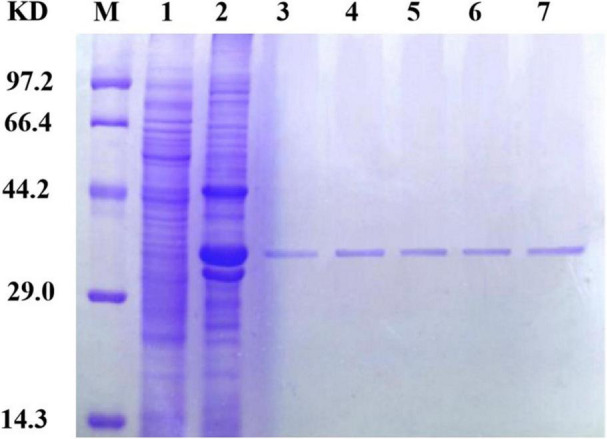
Sip1Aa purification results. Lane M, protein marker; lane 1, pET21b; lane 2, crude protein; lane 3, after purification of Sip1Aa; lane 4, after purification of Sip149-251; lane 5, after purification of Sip153-248; lane 6, after purification of Sip158-243; lane 7, after purification of Sip178-314.

Because different concentrations of imidazole have different impacts on protein purification, including different concentrations in the eluent and binding solutions during protein purification is essential. In this experiment, imidazole was added to the binding solution at concentrations of 10, 20, and 40 mmol/L and to the eluent at concentrations of 40, 250, and 500 mmol/L. The results of SDS-PAGE for the purified protein are shown in Figure 4. Ultimately, a concentration of 40 mmol/L in the binding solution and 250 mmol/L in the eluent were determined to be optimal for SDS-PAGE purification.

### Optimization of Purification Conditions and Determination of Protein Solubility for Sip1Aa and the Mutants

**FIGURE 5:**
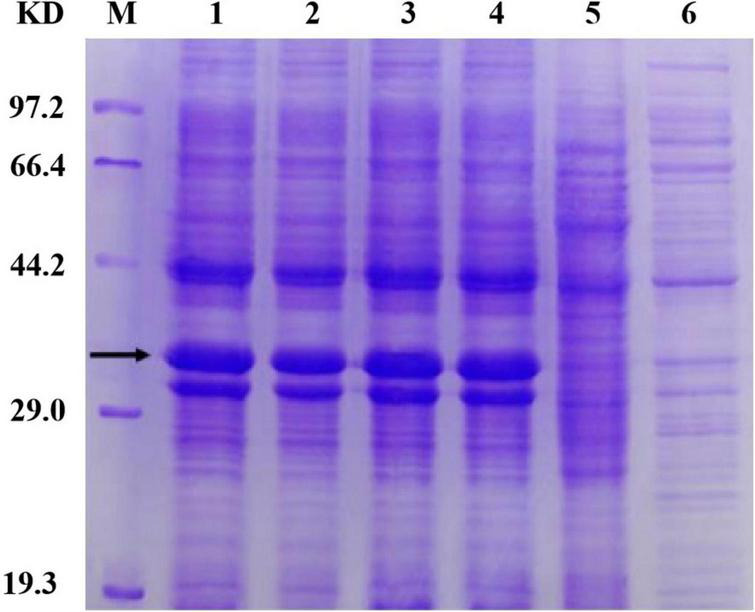
Determination of protein solubility of Sip1Aa and the mutants. Lane M, protein marker; lane 1, Sip149-251; lane 2, Sip153-248; lane 3, Sip158-243; lane 4, Sip178-314; lane 5, Sip1Aa.

The Sip1Aa protein has good insecticidal activity against Coleoptera, but the reason why is unclear. Additionally, it has poor purification effects and low concentrations of purified protein. Therefore, it is essential to obtain high concentrations of purified protein to maximize its insecticidal activity. We found that after repeated rounds of purification and concentration, the concentration of the purified protein increased, the purification effect improved, and protein with a good purification effect and high concentration was obtained. Our work provides new methods of purifying Sip proteins that can provide a strong foundation for subsequent studies. By optimizing the fermentation and induced expression conditions of the mutant protein, we finally determined the expression conditions of the Sip1Aa and mutant proteins, and protein expression was detected with SDS-PAGE. Our work provides new methods of purifying Sip proteins that can provide a strong foundation for subsequent studies. The specific results are shown in Figure 5.

### Determination of Insecticidal Activity

Determination of insecticidal activity Protein expressed in *E. coli* pet 21b was used as a negative control. Uncontaminated organic vegetables were soaked in mutant protein and fed with organic vegetables in mutant protein solution to cultured macaque leaf beetle larvae. The numbers of dead and living insects were counted after 48 h of culture, and the median lethal concentration LC_50_was calculated, and 95% confidence interval regression equation standard error statistical analysis was performed. The results of quantitative tests for insecticidal activity are shown in Table 2. The insecticidal activity of mutants Sip149-251 and Sip178-314 did not change significantly. The insecticidal activity of the mutant Sip153-248 increased 2.76 times, and that of Sip158-243 increased 2.26 times. There was no significant difference in the insecticidal activity of mutant Sip149-251, Sip153-248, Sip158-243, and Sip178-314were against *C. bowringi* Baly compared with that of Sip1Aa protein.

**TABLE 2:** Determination results of insecticidal activity.

Protein	LC_50_ (μg/ml)	95% confidence interval	Regression equation	Standard error	Relative activity values
Sip 1Aa	1.696	1.440–2.121	y = 1.392+0.821	0.138	—
Sip 149-251	1.743	1.479–2.192	y = 1.432+0.822	0.140	1.03↓
Sip 153-248	0.614	0.426–0.791	y = 0.563+0.918	0.108	2.76↑
Sip 158-243	0.751	0.567–0.935	y = 0.675+0.898	0.109	2.26↑
Sip 179-314	1.409	1.228–1.661	y = 1.415+1.004	0.145	1.20↑

### Structural Analysis of Mutation Sites

Structural analyses of the T149, G153, T158, K178, K243, H248, R251, and G314 sites of the Sip1Aa protein were performed with Swiss-Pdb Viewer.

#### Basic Structural Characteristics of Sip1Aa

**FIGURE 6:**
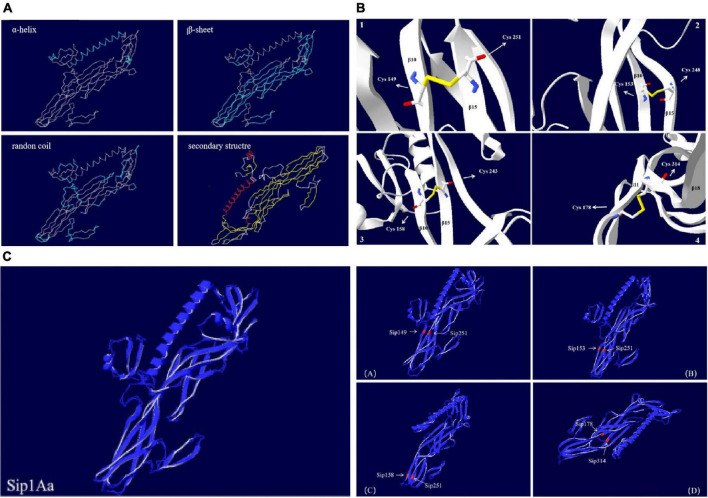
The Sip1Aa protein has the basic structure. **(A)** The basic secondary structure of the Sip1Aa protein. **(B)** Three-dimensional structures of the mutant proteins: (1) Sip149-251, (2) Sip153-248, (3) Sip158-243, and (4) Sip178-314. **(C)** The effect of β-factor on proteins. The Sip1Aa protein and (1) Sip149-251, (2) Sip153-248, (3) Sip158-243, and (4) Sip178-314 proteins. Blue represents temperature, and red represents oscillation.

The Sip1Aa protein is one of many secreted proteins in Bt. It is a member of the Sip family with high virulence and good insecticidal activity. The Sip1Aa protein has the basic structure shown in Figure 6. All the mutation sites in this study are located in the β-sheet, so the mutant protein did not affect its basic secondary structure. Figure 6A shows the secondary structure of the entire Sip1Aa protein in three-dimensional space; it included three α-helices, 21 β-sheets, and 24 random coils. The three-dimensional structures of the mutant proteins are shown in Figure 6B. Mutants Sip149-251, Sip153-243, and Sip153-248 were all located on the β10–β15 loops of Sip1Aa, and mutant 178-314 was located on the β11–β18 loops of Sip1Aa. We performed the effect of temperature on the mutant and Sip1Aa proteins and found that the mutation site changed from stable to mutation site; this is shown in Figure 6C.

**FIGURE 7:**
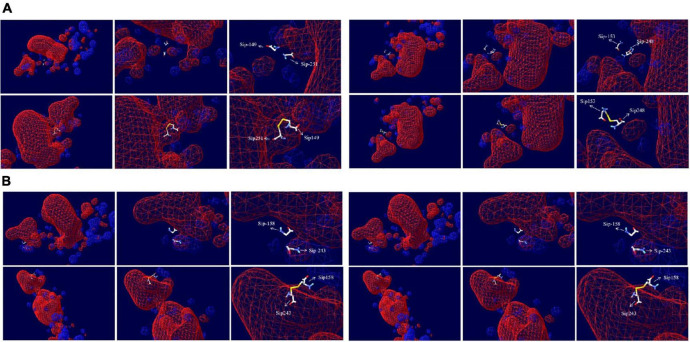
Electrostatic potential spatial distribution of the Sip1Aa protein and **(A)** Sip149-251, Sip153-248, **(B)** Sip158-243, and Sip178-314.

We can see from the spatial distribution of molecular surface Figure 7 that the original Sip149-251 and Sip178-314 were not in the electrostatic field; Sip178 and Sip314 were in the electrostatic field after mutation, and Sip153-248 and Sip158-243 were not in the electrostatic field at any time.

#### Basic Physicochemical Properties of the Mutant Protein

After site-directed mutation, Sip149 changed from two weakly hydrophilic cysteines (–0.7 and –4.5) to highly hydrophilic cysteine (2.5), whereas Sip153 changed from hydrophobic amino acid (–0.4) and hydrophilic amino acid (–3.2) to hydrophilic amino acid (2.5). Sip158 mutated from two hydrophilic amino acids (–0.7 and –3.9) to glycine, and Sip178 changed from hydrophilic amino acids (–3.9 and –3.5) to hydrophilic amino acid (2.5). Table 3 shows the results of basic analyses with ExPASy ProtParam to determine molecular weight, the theoretical isoelectric point, half-life, the instability index, thealiphatic index, and the average hydrophilic value.

**TABLE 3:** Basic Physicochemical Properties of Sip1Aa and Mutant Proteins.

Different kinds of proteins	Molecular weight	Theoretical pI	Estimated half-life	Instability index	Aliphatic index	Grand average of hydropathicity (GRAVY)
Sip	41205.38	6.61	>10 h	24.94	71.24	−0.513
Sip-149-251	41154.37	6.33	>10 h	24.83	71.24	−0.485
Sip-153-248	41217.47	6.54	>10 h	26.04	71.24	−0.490
Sip-158-243	41182.38	6.33	>10 h	24.70	71.24	−0.487
Sip-179-314	41126.43	6.33	>10 h	24.97	71.24	−0.488

**FIGURE 8:**
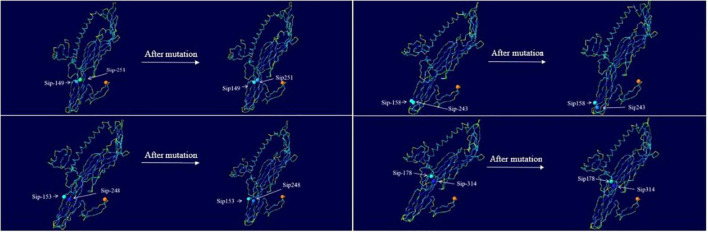
Variation in and spatial distribution of hydrophilicity and hydrophobicity at the mutation sites. Sites with less contact with solvent are shown in blue, and those with more contact are shown in red.

Change in the hydrophilicity and hydrophobicity as well as the spatial distribution was also predicted (Figure 8). The hydrophilicity and hydrophobicity of the original mutant Sip149-251 and mutant protein Sip149-251 did not change significantly compared to the mutation site. The hydrophobicity of the Sip248 mutation site increased when the original mutant Sip153-248 was compared to the mutant protein Sip153-248. The hydrophilicity and hydrophobicity of the mutant site did not change significantly when the original mutant Sip178-314 was compared to the mutant protein Sip178-314.

## Discussion

### The Purification Effect of Purified Protein

Sip proteins are prone to forming inclusion bodies during expression, which makes their extraction challenging. When the number of disulfide bonds is increased, high expression can be achieved and the number of insoluble inclusions can be reduced. As shown in Figure 4, the band of the mutant located at the protein position of interest is thicker than that of Sip1Aa, which demonstrates the increased solubility of Sip proteins. Moreover, no other bands are found after improvement, which demonstrates the high purification efficacy of this method. This method provides technical support for mechanistic studies of Sip insecticidal proteins and for further improving the commercialization of Sip insecticidal protein sent, as shown in Figure 4, which demonstrates the high purification efficacy of this method. This method provides technical support for mechanistic studies of Sip insecticidal proteins and for further improving the commercialization of Sip insecticidal proteins.

### The High Insecticidal Activity of the Sip1Aa and Mutant Proteins

In this study, we selected four pairs of sites for mutations, introduced new disulfide bonds, and successfully expressed the 37.6 kDa soluble protein. This indicates that these amino acid residues were replaced by cysteine without destroying the advanced structure of the protein. Four mutants were constructed: Sip149-251, Sip153-248, Sip158-243, and Sip178-314.

#### Basic Structural Characteristics of the Sip1Aa and Mutant Proteins

The basic structures of the Sip1Aa and mutant proteins included α-helices, β-sheets, and random coils, with no significant difference among them, which demonstrates that the mutant protein did not change the basic structure of the original protein, as mutually verified by the original intention of our design, but still had high insecticidal activity (Wang et al., 2020). When we compared the effect of β-factor compared to Sip1Aa, we found that the mutant protein was less wobbly under the temperature effect and that the temperature affected only the stability of a single mutation site of the mutant, with no significant effect on the stability of the overall mutant protein. Additionally, the overall stability of the mutant protein increased, which suggests that the mutant protein is not sensitive to temperature changes and has increased stability.

#### Basic Physicochemical Properties of the Mutant Protein

The theoretical isoelectric point of the mutant protein was lower than that of the original protein, whereas the pH of the midgut of the great ape leaf beetle was approximately 7.8. After the mutation, the original amino acid mutations of the protein were cysteines. The insecticidal activity of the mutant proteins Sip149-251 and Sip178-314 did not change significantly, and the theoretical isoelectric points of the mutant sites were < 7.8: Sip1Aa was 6.61, mutant Sip153-248 changed to 6.54, and Sip158-243 changed to 6.33 with increased activity; Sip149-251 and Sip178-314 changed to 6.33 with little change in activity. Therefore, Sip153-248/Sip158-243 may be the key site (Zhuo et al., 2009; Kasahara et al., 2021).

The instability index of Sip153-248 was approximately 24, yet it belonged to the stable protein. Therefore, the activity of the protein might increase, resulting in an increase in the instability index, but remain in the stable range (Zhou et al., 2011).

Site-directed mutagenesis was performed on the protein to change the amino acids of the original protein from hydrophobic and weakly hydrophilic to hydrophilic to increase the hydrophilicity of the protein. This increased protein solubility and insecticidal activity, with little effect on the activity and function of Sip149-251 and Sip178-314. The insecticidal activity of the Sip153-248 and Sip158-243 mutants changed markedly, with solubility increasing 2–3-fold with increased stability, especially for the mutation of Sip153-248 from hydrophobic amino acids to hydrophilic cysteine, resulting in increased activity. The increased activity of Sip158-243 suggests that the change from a weakly hydrophilic amino acid to a highly hydrophilic amino acid has a greater impact (Ahamad et al., 2021) on the properties and function of the protein. Therefore, we speculate that this site may be a key site for insecticidal activity. Because the pH of the midgut is alkaline, and the isoelectric point of cysteine is approximately 5.0, the increased insecticidal activity under alkaline conditions may be attributable to the residues of free amino acids with an ionization degree being greatly enhanced compared to the original, which increases dissolved proteins, greatly increases the solubility of insecticidal proteins, and increases dissolved proteins, thereby enhancing the insecticidal effect (Liu and Beveridge, 2002).

#### Comprehensive Structural Analysis of Mutation Sites

In this study, we designed mutation sites in non-conserved regions to enhance activity based on ensuring basic insecticidal activity then selected eight mutation sites to obtain four mutant proteins and ultimately two highly active proteins.

The midgut pH of gibbon leaf beetles is approximately 7.8, and after site-directed mutagenesis, the amino acid mutations at specific sites of the protein were all cysteines. The biological activity, basic physicochemical properties, and molecular characteristics of these four mutant proteins were investigated, and ultimately two mutant proteins with high insecticidal activity were identified. The two mutants had low isoelectric points, high hydrophilicity, and similar basic structures.

The insecticidal activity of the protein improved greatly because although the activity of the protein molecule increased, the basic structure did not change; hence, the basic function of the protein remained the same. Furthermore, its structural stability was enhanced. Therefore, with no change in the basic structure, protein solubility increased along with the solubility of insecticidal proteins, the concentration of soluble proteins, and insecticidal activity. In addition, we speculate that the enhanced functionality of the mutant protein was due in part to the fact that Sip149-251 and Sip178-314 were hydrophobic amino acids from hydrophilic amino acids; Sip251 was a very hydrophilic amino acid, whereas Sip153-248 and Sip158-243 were not hydrophobic amino acids. We speculate that the increased activity may be due to the transformation from hydrophilic amino acids to hydrophobic amino acids, usually located inside the active protein and in the active site, such that the active site is protected by disulfide bonds, which enhances the stability of the mutant protein and its activity (Wu et al., 2013). In addition, Sip149-251 and Sip178-134 mutated from the original amino acid sites were not bound to the sites with stable disulfide bonds, which enhanced stability but reduced insecticidal activity. Sip153-248 and Sip158-243 mutated from the original hydrogen bond to the present disulfide bonds had enhanced structural stability, increased protein solubility (Wu et al., 2021), decreased inclusions, and 2.76-fold increased insecticidal activity. The chemical bond changed from hydrogen to disulfide. The hydrophobicity also changed from the original hydrophilic amino acid to the hydrophobic amino acid, such that the key active sites were no longer in the reducing environment (Bechtel and Weerapana, 2017), which enhanced protein stability. Compared to the original protein, the mutant protein is easier to ionize, which results in good solubility and better activity, but after cysteine mutation, the insecticidal activity of Sip149-251 and Sip178-314 did not change significantly; therefore, we speculate that Sip149-251 and Sip178-314 may not be the key active sites of this insecticidal protein. Protein foldingis the result of various thermodynamic interactions. In most proteins, the amide bond linking the amino acid residues on the main chain is the only covalent bond. In secreted proteins or extracellular portions of cell surface proteins, additional covalent bonds in the form of disulfide bridges may exist between the side chains of cysteine residues because of the lack of exposure to the intracellular reducing environment (Beyer et al., 2018).

In the future, we consider adding these eight cysteine mutation sites simultaneously on a mutant protein to observe the changes in its insecticidal activity, protein structure, etc. At the same time, although Sip 1Aa insecticidal protein showed good insecticidal activity, the study of insecticidal site and insecticidal mechanism was relatively blank. In this experiment, on the basis of obtaining high concentration insecticidal protein, His-Pull Down and yeast two-hybrid will be used to screen potential receptor proteins. The receptor proteins were finally screened by GST-Pull Down and RNAi, BiFC *in vitro* and *in vivo* validation, and other techniques for validation. These results show that site-directed mutagenesis is of great significance in the study of insecticidal activity (Yang et al., 2021).

## Data Availability Statement

The original contributions presented in the study are included in the article/Supplementary Material, further inquiries can be directed to the corresponding author/s.

## Author Contributions

LW and J-GG: conceptualization. LW: methodology, software, formal analysis, investigation, data curation, writing—original, and draft preparation. M-YD and JW: validation. H-TL: resources, writing—review, and editing. LW and R-ML: visualization. J-GG: supervision, project administration, and funding acquisition. All authors have read and agreed to the published version of the manuscript.

## Conflict of Interest

The authors declare that the research was conducted in the absence of any commercial or financial relationships that could be construed as a potential conflict of interest.

## Publisher’s Note

All claims expressed in this article are solely those of the authors and do not necessarily represent those of their affiliated organizations, or those of the publisher, the editors and the reviewers. Any product that may be evaluated in this article, or claim that may be made by its manufacturer, is not guaranteed or endorsed by the publisher.

## Footnotes

1
http://www.sbg.bio.ic.ac.uk/phyre2/html/page.cgi?id=index

2
https://zhanggroup.org//ModRefiner/

3
https://www.expasy.org/

4
http://cptweb.cpt.wayne.edu/DbD2/

5
http://swissmodel.expasy.org/
