# Supplementary figures and images for: Effects of Site-Directed Mutagenesis of Cysteine on the Structure of Sip Proteins
Source: Front Microbiol. 2022 Apr 29;13:805325. doi: 10.3389/fmicb.2022.805325 (PMC9100928; doi:10.3389/fmicb.2022.805325)

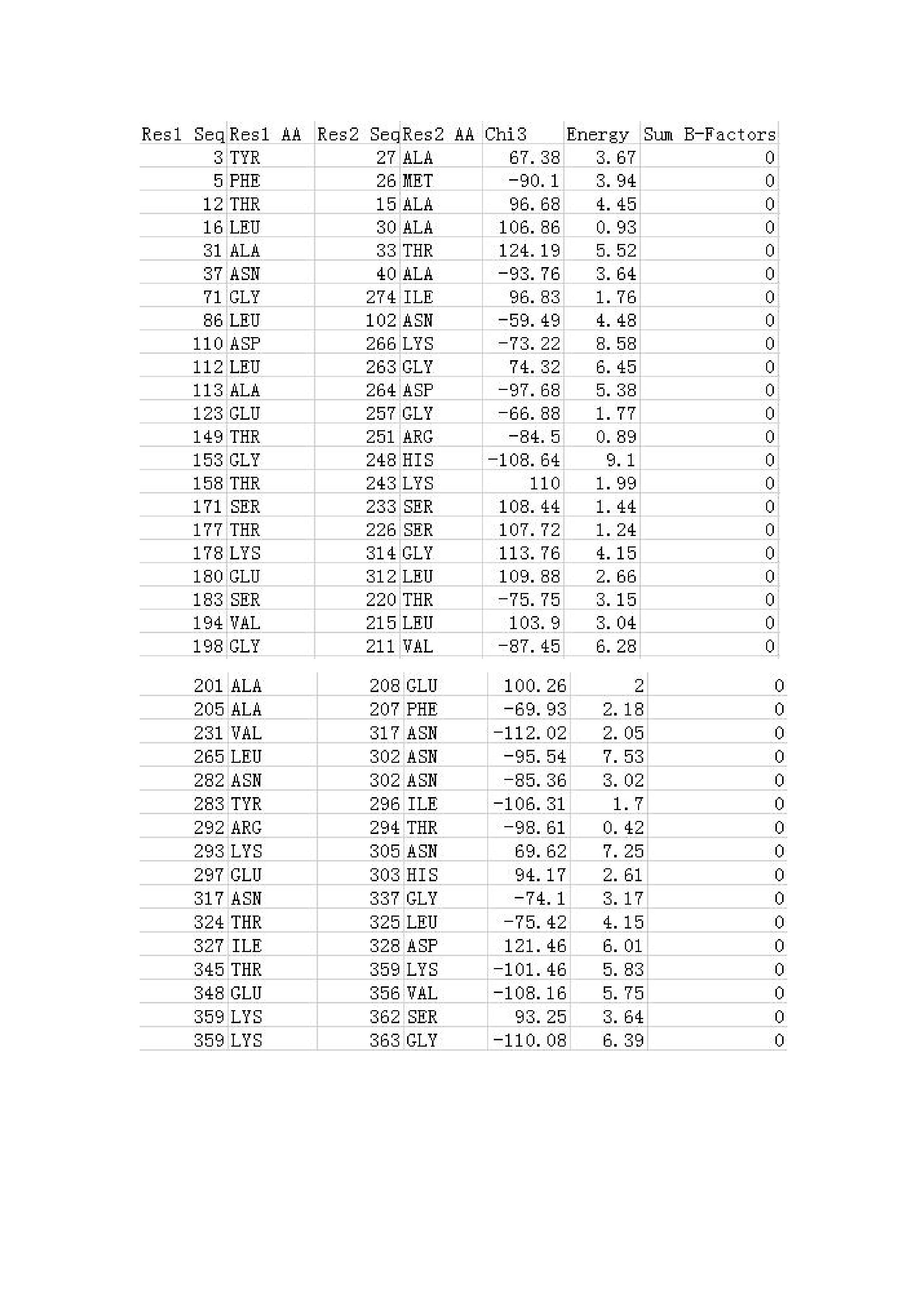

Supplement: Supplementary file 1 [file Image_1.jpeg]

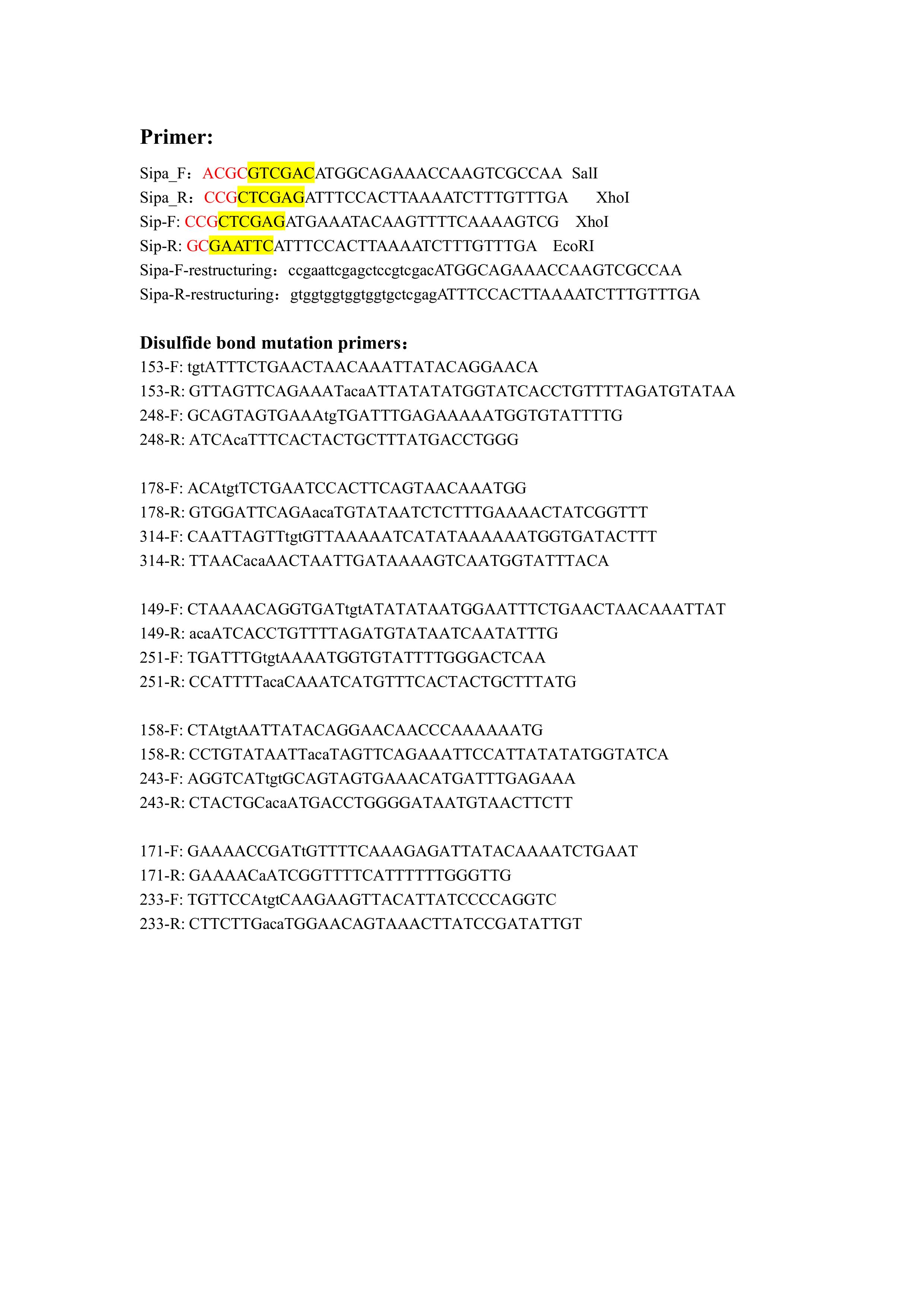

Supplement: Supplementary file 2 [file Image_2.jpeg]

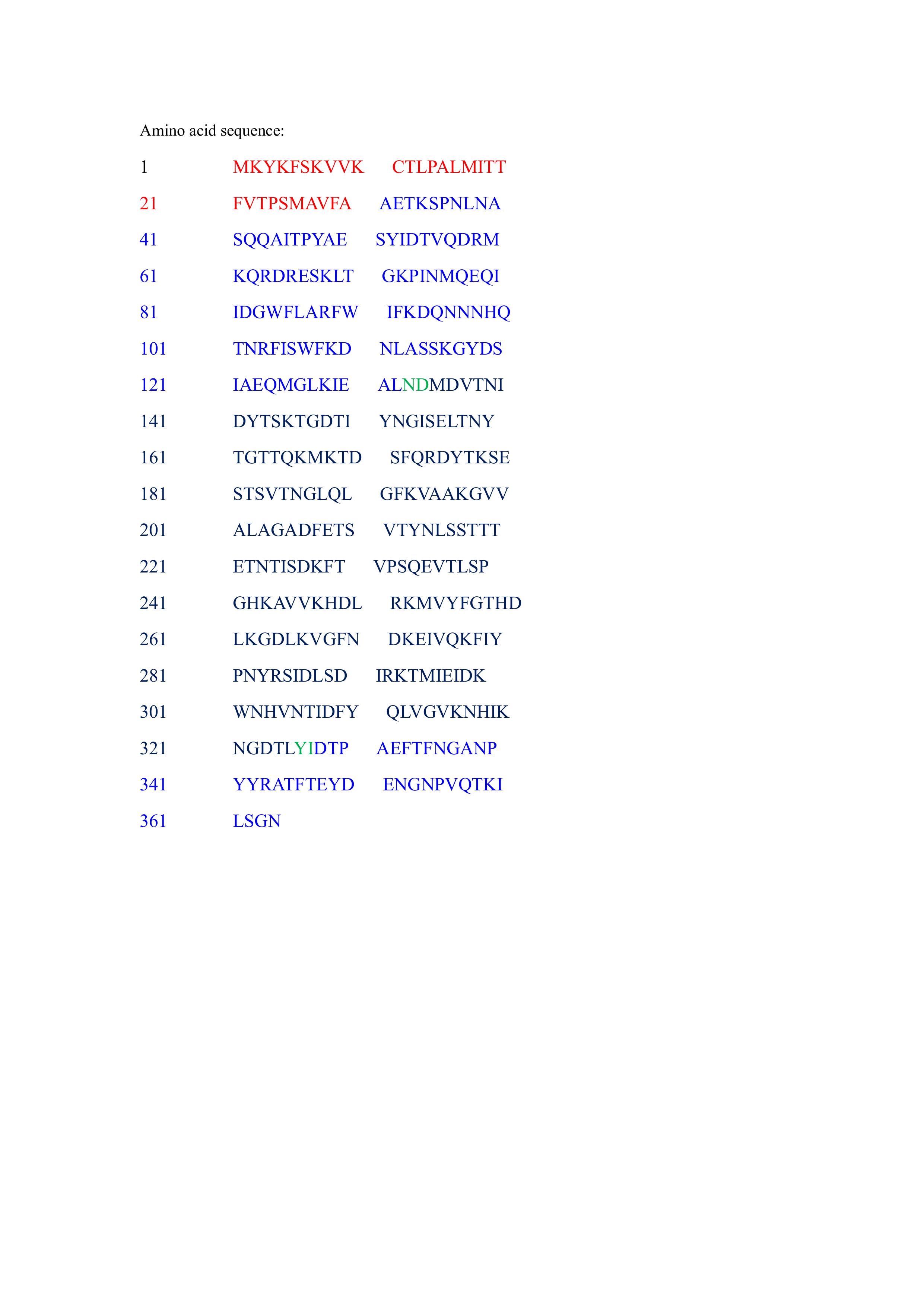

Supplement: Supplementary file 3 [file Image_3.jpeg]
